# Gonorrhea and Marriage

**Published:** 1913-12

**Authors:** William J. Robinson

**Affiliations:** New York, Chief of the Department of Genito-Urinary Diseases and Dermatology, Bronx Hospital and Dispensary; Editor Collectanea Jacobi, Etc.


### Gonorrhea and Marriage.

BY WILLIAM J. ROBINSON, M. D., NEW YORK,
Chief of the Department of Genito-Urinary Diseases and Dermatology, Bronx Hospital and Dispensary; Editor *Collectanea Jacobi*, etc.

There is no question which the genito-urinary specialist is called upon to answer more frequently, and which gives him more painful concern, than the question of a gonorrheic or ex-gonorrheic: "Doctor, is it safe for me to get married?" I have had an unusually large share of such cases to consider, and I have not had occasion to regret the advice which I gave, nor, as far as I know, has the patient—if he followed it.

It need not be repeated here that I disagree with my overzealous and sensational friends who like to assert that "once a gonorrhea always a gonorrhea," and who would forbid the man who once had the misfortune to harbor the gonococcus, even for ever so short a time, ever to marry. If we were to apply this injunction in practice, the world's population would in a short time become pretty well thinned out and the number of unmarried women would increase a thousandfold. Such severity against those once gonorrheally infected is not only cruel, it is unnecessary, because it is false and has no justification in fact. That many men who consider themselves cured not infrequently infect their wives is as well known to me as to anybody. But it is absurd and pernicious to go to extremes in the matter. In medicine as elsewhere common sense must not be dispensed with.

Can we tell when a gonorrhea is perfectly cured? Yes. If a man has been free from any discharge whatever for a month, while living his regular life; if his urine, particularly his morning urine, is free from shreds, or contains only small particles of shreds, which are free from gonococci; and if three expressed samples of secretion from the prostate, taken at different times, are free from gonococci, then the patient is *cured*. Does that mean that we can assert positively that the man does not harbor a single gonococcus? No. But it is not necessary. A person who harbors a tubercle bacillus, but is otherwise perfectly well, does not suffer from tuberculosis, and is not a dangerous individual. We cannot assert about any person with absolute positiveness that he does not harbor some germs of some kind, but that does not render that individual dangerous.

To come now to the definite practical points. When a man answers the requirements outlined above, we have no hesitation in permitting marriage. But suppose a man does have a scanty discharge or a drop in the morning, or a few shreds? If they are free from gonococci, we also give permission, though we advise caution, that is, to be moderate in intercourse and to have the wife take a mild antiseptic vaginal douche after coitus.

If the scanty discharge, or the shreds, or the expressed prostatic discharge contains a few gonococci, what then? Then we refuse permission. But it sometimes happens that all arrangements have been made for the wedding, and then only the man comes to be examined. He should have had himself examined before he made the arrangements for the wedding, but he hasn't, and that's all there is to it, and you cannot undo the past. We examine him and do find gonococci. To put off the wedding would lead to a great trouble and perhaps to scandal. He begs that something be done for him, to help him out of the difficulty. We may do it reluctantly, but we generally help the patient out. An antiseptic vaginal suppository and some antiseptic tablets, are prescribed, with the directions that the wife use a suppository a few minutes before intercourse and an injection after. This course has been pursued in a number of instances, and in not a single case has infection resulted. The husband in the meantime took treatment until he was cured or practically cured.

Sometimes a man comes to you in the acute stage of gonorrhea, with profuse discharge, and tells you he is going to get married in three or four days. He was all right, but three or four nights ago, on the occasion of his bachelor dinner, he "went out with the boys," and here is the result. By the use of condoms it would probably be possible to avoid infection, but a man in the acute stage of gonorrhea should not get married, and we would not be a party to such iniquity. He has to invent some excuse and postpone the wedding for a month or two.

One thing we must not forget: That a urethra which has even completely recovered from a long-standing gonorrhea is a *locus resistentie minoris,* does not possess the same resisting power that a normal urethra does, and may therefore more readily become infected with ordinary bacteria, which are often present in the vaginas of women who are not in the habit of taking frequent vaginal douches. This is particularly the case with virgins who, for fear of rupturing the hymen, take no douches, often even when suffering with leucorrhea in an aggravated degree. We must also remember that defloration frequently causes a real wound or wounds, which may suppurate for a few days. Take then such a case of a pure, chaste girl who is suffering with leucorrhea, and who has developed a slightly suppurating wound. The bridegroom, who has been assured that he is perfectly cured, but who has not been warned that his urethra is and may forever remain rather susceptible and that a certain amount of care is always in order, indulges to excess, as every bridegroom thinks he is morally bound to do, and in a few days he develops a simple bacterial urethritis, which he mistakes for gonorrhea—to the layman all urethral discharges mean gonorrhea—and in anguish and distress he runs to the doctor to take him to task for misleading him, for telling him that he was cured, when as a matter of fact he just got a relapse. A microscopic examination and three or four injections of potassium permanganate or weak silver nitrate solution (1:1000) makes matters right again. To avoid any such unpleasant contingencies, we always consider it imperative to explain to every discharged-as-cured patient the true state of affairs, especially if he is going to get married, so that he may be somewhat on his guard, both as to cleanliness and as to excess.

### LONG DORMANT CASES OF GONORRHEA.

A word about the cases of long dormant gonorrhea. We hear stories of men who had suffered with gonorrhea, had been pronounced cured, had married, lived with their wives five or eight or ten years without any trouble, and then all at once the dormant gonococci woke up, the man got the disease and then promptly infected his wife. We remember one case in which the gonorrhea was said to have been dormant for twenty years before it woke up! Let me say that I take all such stories not with a grain but with a gram of salt. I do not believe them. Of course such things may be possible. Everything is possible in medicine. But there is a gulf between what is possible and what is probable. I assert that there is not a single such well authenticated and unimpeachable case on record. If we looked more closely into some of the cases, we would find that the so-called relapse is not a relapse, but a new infection obtained in the ordinary way. It is natural that when a "good" husband strays from the safe but narrow path of monogamy and gets a gonorrhea, he should wish to persuade his wife and even his doctor that his disease is but the awakening of an ancient trouble contracted in antenuptial days, but we are fully justified in maintaining a skeptical though respectful attitude towards all such stories. The gonococcus is a hardy germ, but he does not hibernate for a quarter of a century, nor for a tenth of a century, to be suddenly awakened to pernicious activity. Several interesting cases could be related which would throw some light on this mooted subject, but one case will suffice:

A. A. came to be treated for a relapse of an old clap. He is a big strapping policeman, has been married seven years and perfectly well all the time. About three years before marriage he had a severe gonorrhea, of which he was, however, perfectly cured. His present condition presented all the symptoms of a fresh, newly acquired attack of gonorrhea. But when questioned as to the possibility of it being a new gonorrhea, he made a stout denial. He was even indignant. No, since his marriage he settled down, and never had anything to do with any woman except his wife. About ten days later, not quite satisfied with the progress of his disease, he rather sheepishly asked me if a new gonorrhea and a relapse of an old one required different methods of treatment. I told him, of course they did, but said nothing further. At the next visit he finally came out with the statement that he feared it might be a new attack, as recently he had been indulging rather freely in extra-matrimonial relations.

And I am sure that very many if not all of those wonderful cases of long, long dormant and suddenly awakened gonorrheas would be found to have as simply an etiology as our policeman's did. That a gonorrhea may remain infectious for many, many years is of course admitted. But then it is an *uncured* gonorrhea, a gonorrhea which gives some symptoms. And this is an entirely different matter from the subject under discussion.—*Med. Review of Reviews.*

